# Parallel improvement of left ventricular geometry and filling pressure after transcatheter aortic valve implantation in high risk aortic stenosis: comparison with major prosthetic surgery by standard echo Doppler evaluation

**DOI:** 10.1186/1476-7120-11-18

**Published:** 2013-06-03

**Authors:** Marco Fabio Costantino, Maurizio Galderisi, Ernesta Dores, Pasquale Innelli, Giandomenico Tarsia, Maurilio Di Natale, Ciro Santoro, Francesco De Stefano, Roberta Esposito, Giovanni de Simone

**Affiliations:** 1 Division of Cardiology, San Carlo Hospital, Potenza; 2 Division of Cardioangiology with CCU, Department of Translational Medical Sciences, Federico II University Hospital, Naples, Italy; 3 Naples Division of Cardiology, Federico II University Hospital, Naples, Italy; 4 Villa d’Agri Hospital, Villa d’Agri, Potenza, Italy

**Keywords:** Transcatheter aAortic valve implantation, Doppler echocardiography, Relative wall thickness, Left ventricular mass

## Abstract

**Purpose:**

The effect of Transcatheter Aortic Valve Implantation (TAVI) on left ventricular (LV) geometry and function was compared to traditional aortic replacement (AVR) by major surgery.

**Methods:**

45 patients with aortic stenosis (AS) undergoing TAVI and 33 AVR were assessed by standard echo Doppler the day before and 2 months after the implantation. 2D echocardiograms were performed to measure left ventricular (LV) mass index (LVMi), relative wall thickness (RWT), ejection fraction (EF) and the ratio between transmitral E velocity and early diastolic velocity of mitral annulus (E/e’ ratio). Valvular-arterial impedance (Zva) was also calculated.

**Results:**

At baseline, the 2 groups were comparable for blood pressure, heart rate, body mass index mean transvalvular gradient and aortic valve area. TAVI patients were older (p<0.0001) and had greater LVMi (p<0.005) than AVR group. After 2 months, both the procedures induced a significant reduction of transvalvular gradient and Zva but the decrease of LVMi and RWT was significant greater after TAVI (both p<0.0001). E/e’ ratio and EF were significantly improved after both the procedure but E/e’ reduction was greater after TAVI (p<0.0001). TAVI exhibited greater percent reduction in mean transvalvular gradient (p<0.05), Zva (p<0.02), LVMi (p<0.0001), RWT (p<0.0001) and E/e’ ratio (p<0.0001) than AVR patients. Reduction of E/e’ ratio was positively related with reduction of RWT (r = 0.46, p<0.002) only in TAVI group, even after adjusting for age and percent reduction of Zva (r =0.43, p<0.005).

**Conclusions:**

TAVI induces a greater improvement of estimated LV filling pressure in comparison with major prosthetic surgery, due to more pronounced recovery of LV geometry, independent on age and changes of hemodynamic load.

## Introduction

Aortic stenosis (AS) is the most common valvular heart disease in western countries [1]. It induces pressure overload of the left ventricle, causing left ventricular (LV) hypertrophy (LVH) associated with abnormalities of systolic and diastolic function, recognized risk factors for cardiac morbidity and mortality [2-6]. The number of patients with associated co-morbidities and high operative risk is increasing [7]. Transcatheter aortic valve implantation (TAVI) is a promising therapy for AS in these high risk patients [8,9]. Several recent studies have shown feasibility and safety of TAVI in short and mid-term follow-up periods [10-15]. Survival in patients with severe AS who cannot undergo surgery has been improved by TAVI [15,16]. The early results are encouraging, with reported 30-day mortality rates below 10% and 1-year survival rates above 70% [15,17-22].

Recent observations have shown how TAVI could be followed by an immediate decrease in transaortic pressure gradient and a consequent reduction in LV afterload [23,24]. Although the reduction of LV mass and the improvement of LV diastolic function have been demonstrated to take place early after TAVI [25-28], it is still unclear whether this recovery is of similar magnitude of that obtained with open-chest aortic valve replacement (AVR). Accordingly, the objective of our study was to compare effects of TAVI and traditional AVR on LV geometry and function using standard Doppler-echocardiography, over 2-months of follow-up.

## Methods

### Study population

The study population included consecutive patients with symptomatic severe AS and high risk [29], who underwent baseline standard transthoracic echo-Doppler exam between November 2011 and July 2012 at San Carlo Hospital (Potenza) and repeated echo Doppler exam 2 months after the respective procedure (TAVI or AVR). The high risk was established according to the calculated Society of Thoracic Surgeons (STS) score > 10% [30].

Exclusion criteria included a bicuspid aortic valve disease (n=1) , previous acute myocardial infarction (n=3), significant coronary artery disease requiring revascularization (n=2), LV ejection fraction ≤ 30% (n=5), severe mitral or aortic valve regurgitation (n=3), atrial fibrillation (n=3), transient ischemic attack or stroke within the previous 6 months (n=1), and severe renal insufficiency (estimated GFR <30 ml/m^2^).

After exclusions of 18 patients, echo Doppler data of 45 TAVI patients (26 Edwards Sapien XT and 19 Medtronic CoreValve and 33 AVR patients (Carpentier Edwards SVA) were collected and compared. Written informed consent was obtained from each patient. TAVI and AVR were performed according to the respective standardized procedures [31-33]. Patients undergoing TAVI were not considered suitable candidates for open-chest surgery because they had coexisting conditions that would be associated with a predicted probability of 50% or more of either death by 30 days after surgery or a serious irreversible condition [34]. All patients had NHYA class II, III, or IV symptoms.

### Procedures

#### Standard echocardiographic examination

Doppler echocardiographic exams were performed using a Vivid 7 ultrasound scanner (GE, USA) equipped with a 2.5 MHz phased-array transducer according to the standards of our laboratory [35,36]. All echocardiograms were read off-line by an experienced fellow (ED) at the Echocardiography Laboratory of Department of Translational Medical Sciences of the Federico II University Hospital in Naples, Italy, under the supervision of a senior attending cardiologist (MG). Diagnosis and severity of aortic valve stenosis was performed by measuring peak and mean transvalvular aortic gradient and aortic valve area (AVA) computed by the continuity equation. Aortic stenosis was defined severe when mean transvalvular gradient was > 40 mmHg. Standard linear measures were taken to compute LV mass that was normalized for height powered to 2.7 (LVMi) [37]. LV hypertrophy (LVH) was defined as LVMi ≥ 45 g/m^2.7^ in women and ≥ 49 g/m^2.7^ in men. Two-dimensional LV end-diastolic and end-systolic volumes were measured by the modified Simpson method (average of apical 4- and 2-chamber views) and ejection fraction (EF) was calculated. Left atrial (LA) volume was assessed by the biplane area-length method from the apical approach, taking care to obtain multiple, dedicated views of the left atrium purposely oriented to maximize LA area and optimal definition of LA wall, according to a standardized method [38]. LA volume was indexed for body surface area (left atrial volume index = LAVi). Stroke volume (ml) was calculated by pulsed Doppler method of LV outflow tract and indexed for body surface area (stroke volume index, ml/m^2^). Transmitral pulsed Doppler was recorded in the apical 4-chamber view. Early (E) and atrial (A) peak velocities (m/sec) and their ratio, and E velocity deceleration time were measured. By pulsed Tissue Doppler, early diastolic velocity (e’) was measured in apical 4-chamner view at the lateral mitral annulus. Attention was paid to the Doppler spectral gain settings and the velocity scale was kept at about 20 cm/s above and below the baseline. Minimal angulation (<20°) was maintained between the ultrasound beam and the plane of cardiac motion during the sampling of the lateral mitral annular site. The ratio of transmitral peak E velocity to peak e’ velocity was calculated as an estimate of LV filling pressure by using e’ velocity of lateral mitral annulus (E/e’ ratio). Valvular arterial impedance (Zva) was determined as an index of global LV load according to the formula = (ΔP + systolic BP)/SVI where ΔP = mean transvalvular systolic pressure gradient, BP = blood pressure and SVI = stroke volume index [39].

### Statistical analysis

Statistical analysis was performed by SPSS package, release 12 (SPSS Inc., Chicago, Illinois, USA). Data are presented as mean value ± SD. Intergroup comparison at baseline was obtained by one-factor ANOVA. The comparison of data before and after surgery (time) and the impact of the procedure (treatment: TAVI vs. AVR) was be statistically assessed using a 2-way ANOVA for repeated measures. Least squares linear regression was used to evaluate univariate correlates of a given variable. The null hypothesis was rejected at p ≤ 0.05.

## Results

The main clinical characteristics and main echo Doppler features of the 2 study groups at baseline are reported in Table 1. The TAVI group was older than the AVR group, with similar body mass index, blood pressure and heart rate. TAVI had higher baseline LVMi than AVR, with comparable transvalvular mean gradient and AVA, Zva, relative diastolic wall thickness, EF, E/e’ ratio and LAVi. The prevalence of LVH was 100% (45/45) in the TAVI group and 91 % (30/33) in the AVR group (data not reported in Table).

**Table 1:** Characteristics of the 2 study groups at entry

**Variables**	**TAVI**	**AVR**	**p**
	**(n=45)**	**(n=33)**	
Sex (M/F)	25/20	18/15	0.929
Age (years)	80.8 ± 6.2	74.9 ± 4.6	<0.0001
Height (m)	1.63 ± 0.08	1.64 ± 0.05	0.423
Weight (Kg)	66.2 ± 10.3	67.1 ± 9.0	0.694
BSA	1.71 ± 0.16	1.73 ± 0.13	0.554
BMI (kg/m^2^)	24.8 ± 3,0	24.7 ± 2.3	0.936
Systolic BP (mmHg)	125.6 ± 12.9	129.9 ± 11.3	0.131
Diastolic BP (mmHg)	72.3 ± 9.8	73.7 ± 10.7	0.536
HR (beats/min)	71.9 ± 8.6	73.3 ± 8.1	0.461
STS Score (%)	14.7 ± 3.4	13.8 ± 2.8	0.211
Combined MR (%)	53.3	57.6	0.802
Combined AR (%)	53.3	54.5	0.830
TG max (mmHg)	88.3 ± 14.6	94.1 ± 14.6	0.087
TG mean (mmHg)	48.5 ± 8.8	50.6 ± 7.2	0.255
AVA (cm / m ^2^)	0.62 ± 0.11	0.61 ± 0.10	0.541
Zva (mmHg/m x m ^2^)	5.1 ± 1.3	5.5 ± 1.9	0.317
LVMi (g/m ^2.7^)	80.0 ± 17.9	69.2 ± 15.8	<0.01
RWT	0.56 ± 0.09	0.54 ± 0.08	0.353
EF (%)	52.1 ± 6.9	55.3 ± 8.0	0.063
SVi (ml/m^2^)	46.8 ± 12.5	43.4 ± 11.9	0.232
E/e’ ratio	11.9 ± 2.9	12.3 ± 2.8	0.537
LAVi (ml/m^2^)	34.5 ± 7.7	36.2 ± 3.6	0.214

AR = Aortic regurgitation, BSA = Body surface area, BMI = Body mass index, BP = Blood pressure, E = Transmitral early diastolic velocity, e’ = early diastolic velocity of mitral annulus , EF = Ejection fraction, HR = Heart rate, HTN = Arterial hypertension, LAVi = Left atrial volume index, LVMi = Left ventricular mass index, MR = Mitral regurgitation, RWT = Relative wall thickness, STS = Society of Thoracic Surgeons, SVi = Stroke volume index, TG = Transvalvular gradient.

Table 2 shows the comparison of echo Doppler variables before and after the procedures and impact of the procedure (TAVI vs. AVR). Both the procedures induced a significant reduction of transvalvular gradient and Zva but the decrease of LVMi and relative wall thickness was significantly greater after TAVI. EF was increased and E/e’ ratio was reduced after either procedures but the E/e’ ratio reduction was significantly greater after TAVI than after AVR.

**Table 2:** Comparison of echo Doppler variables before and after the procedures and impact of the procedure (TAVI vs. AVR)

**Variables**	**At entry**	**TAVI**	**At entry**	**AVR**	**Within patients difference**	**Time related difference between procedures**
HR (beats/m)	71.8 ± 8.6	70.9 ± 7.8	73.3 ± 8.1	73.6 ± 9,3	0.706	0.381
TG max (mmHg)	88.3 ± 14.6	21.3 ± 7.7	94.1 ± 14.6	26.9 ± 5.6	<0.0001	0.950
TG mean (mmHg)	48.5 ± 8.8	11.1 ± 4.2	50.6 ± 7.2	14.0 ± 3.6	<0.0001	0.570
Zva (mmHg/m x m ^2^)	5.1 ± 1.3	3.9 ± 1.1	5.5 ± 1.9	4.5 ± 1.6	<0.0001	0.225
LVMi (mg/m^2.7^)	80.0 ± 17.9	70.6 ± 15.7	69.2 ± 15.8	68.7 ± 15.3	<0.0001	<0.0001
RWT	0.56 ± 0.09	0.50 ± 0.08	0.54 ± 0.08	0.54 ± 0.08	<0.0001	<0.0001
EF (%)	52.1 ± 6.9	55.2 ± 8.6	55.3 ± 8.0	57.2 ± 8.8	<0.0001	0.244
SVi (ml/m^2^)	46.8 ± 12.5	46.6 ± 10.7	43.4 ± 11.9	43.8 ± 10.4	0.886	0.725
E/A ratio	1.06 ± 0.55	1.21 ± 0.32	0.96 ± 0.25	1.10 ± 0.17	<0.001	0.981
E velocity DT (ms)	198.8 ± 76.7	187.4 ± 65.8	194.3 ± 65.7	181.0 ± 50.1	<0.01	<0.815
e’ velocity (cm/s)	8.6 ± 1.3	11.6 ± 1,5	8.9 ± 1.3	12.5 ± 1.6	<0.0001	0.147
E/e’ ratio	11.9 ± 2.9	9.06 ± 1.9	12.3 ± 2.8	11.5 ± 2.2	<0.0001	<0.0001
LAVi (ml/m^2^)	34.5 ± 7.7	28.2 ± 6.9	36.2 ± 3.6	27.8 ± 3.5	<0.0001	0.484

A = Transmitral atrial velocity, DT = Deceleration time, E = Transmitral early diastolic velocity, e’ = Pulsed Tissue Doppler early diastolic velocity of the lateral mitral annulus, EF = Ejection fraction, LAVi = Left atrial volume index, LVMi = Left ventricular mass index, RDWT = Relative diastolic wall thickness, SVi = Stroke volume index, TG = Transvalvular gradient, Zva = Valvular-arterial impedance.

**Figure 1:**
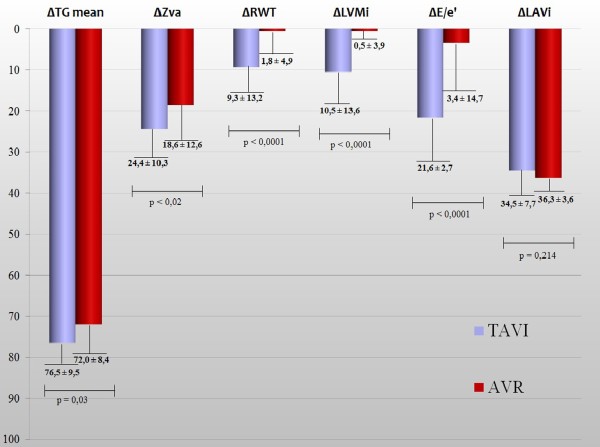
Comparison of per cent reduction of the main echo Doppler parameters in TAVI and AVR groups.

Figure 1 displays the comparison of percent changes of the main echo Doppler parameters between the 2 groups. Percent reductions of mean transvalvular aortic gradient, Zva, LVMi, relative wall thickness and E/e’ ratio were significantly greater in TAVI than in AVR patients.

**Figure 2:**
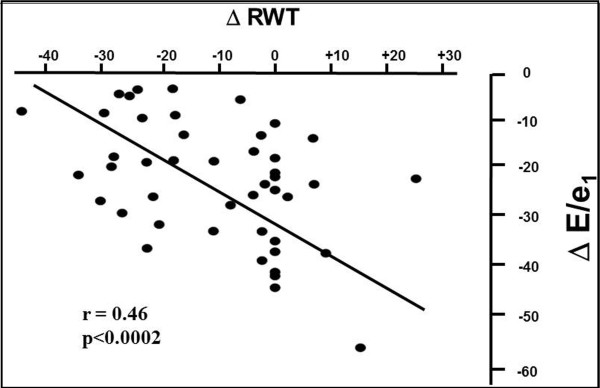
Scatterplot and regression line of the relation between percent reduction of relative wall thickness and percent reduction of LV filling pressure in the TAVI group.

**Figure 3:**
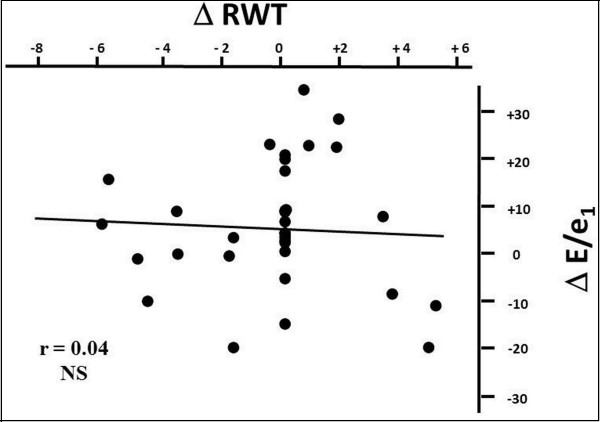
Scatterplot and regression line of the relation between percent reduction of relative wall thickness and percent reduction of LV filling pressure in the AVR group.

Per cent reduction of relative wall thickness was not significantly related to either transaortic mean gradient or Zva in both TAVI (r = 0.22 and r = 0.20, NS) and AVR group (r = 0.24 and r = 0.23, NS). Similarly, the per cent reduction of E/e’ ratio was not significantly related with transaortic mean gradient or Zva in both TAVI (r = 0.10 and r = 0.11, NS) and AVR group (r = 0.13 and r = 0.14, NS). In contrast, the percent reduction of relative wall thickness was positively related to the percent reduction of E/e’ ratio in the TAVI group (r = 0.46, p<0.0002) (Figure 2) but not in AVR patients (r = 0.04, NS) (Figure 3). The relation found in the TAVI group remained significant even after adjusting for age and percent reduction of Zva (r = 0.43, p<0.005). No significant relation was found between percent reduction of LVMi and percent reduction of E/e’ ratio in both TAVI (r = 0.18, NS) and AVR (r = 0.28, NS) groups.

## Discussion

The present study demonstrates that 2 months after replacement of aortic valve for AS, TAVI induces a faster recovery of LV geometry and greater reduction of estimated LV filling pressure in comparison with traditional AVR and that the reduced LV filling pressure is strongly due to changes of the same LV geometry only in TAVI group.

Because of pressure overload, LV structural changes developing in patients with AS are characterized by LV concentric remodeling and concentric LVH. These changes are associated with myocardial interstitial fibrosis, producing LV diastolic abnormalities and left atrial remodeling [40] while systolic chamber dysfunction has a later onset [2-5]. After the substitution of aortic valve, clinical improvement is expected and improved diastolic stiffness and relaxation are observed in late follow-up [4].

Recent studies have demonstrated that TAVI can determine an early regression of LVH and a significant improvement of LV diastolic properties [25-28]. In these studies the immediate reduction of transvalvular pressure gradient was associated with significant reduction of LV mass [27], improvement of diastolic filling pattern [25,26,28], reduction of LV filling pressure [27,28] and decrease in left atrial size [28] while a clear improvement of systolic LV chamber function was observed only after 3 months [26].

To the best of our knowledge, the present study is the first to document that recovery of LV geometry and improvement of LV filling pressure are both more evident 2 months after TAVI than after traditional AVR at the same time. EF improvement was not significantly different between TAVI and AVR group confirming previous results [26]. However, the reduction of both relative wall thickness and E/e’ ratio was more pronounced after TAVI than after AVR while LVMi was significantly reduced only after TAVI. These findings were further reinforced by the observation that the percent reductions of relative wall thickness and E/e’ ratio were substantially greater after TAVI than after AVR.

It is noteworthy that no relation was detected between the percent reduction of transvalvular pressure gradient or Zva and the percent decrease of relative wall thickness or LV filling pressure in TAVI as well as in AVR group. Accordingly, the substantial difference in the recovery of LV geometry after TAVI could not be due to the pure reduction of loading conditions, but should be ascribed to own factors related to the respective surgical procedure. A transient peri-operative LV dysfunction is well recognized after traditional AVR, this effect being related to cardiopulmonary by-pass [41]. This transient functional deterioration is further confirmed by elevated BNP and troponin I serum levels occurring early after AVR [42,43]. In the TAVI procedure, the consequences of cardiopulmonary by-pass are avoided and LV remodeling can occur likely due to less neuro-hormonal stimulus sustaining initial persistence of LVH.

The main finding of the present study is in fact the relation between the percent reduction of relative wall thickness and the estimated LV filling pressure (by E/e’ ratio), found only in the TAVI group. This relation remained significant even after adjusting for age and percent reduction of Zva, an index of LV global load which accounts for the effects of both AS and systemic arterial compliance, is one of the main determinant of exercise capacity [44] and is prognostically validated [42]. In post-cardiac surgery of patients with overall preserved systolic LV chamber function, the degree of E/e’ ratio had been shown to be significantly associated with BNP levels, a finding which indicates left atrial pressure as a major determinant in BNP release in this clinical setting [45]. The results of the present study highlight therefore how the early recovery of LV geometry occurring after TAVI could be as fast as a beneficial effect on the reduction of LV filling pressure and may well explain the evidence of better short and long-term prognosis of patients with AS undergoing TAVI [18-22]. Elevated LV filling pressure is the key determinant of cardiac symptoms and prognosis in patients with chronic heart failure and coronary artery disease, independent on the values of EF [46,47]. One-year antihypertensive therapy resulting in relative wall thickness reduction has been previously found to be associated with significant improvement of LV diastolic filling parameters related to active relaxation and passive chamber stiffness, independent of BP reduction, in hypertensive patients with LVH of the LIFE study [48]. Our results extends these relations to patients with AS undergoing TAVI in a time period which is substantially shorter to that needed by anti-hypertensive drugs to achieve the same effect in arterial systemic hypertension.

### Limitations of the study

The main limitation of the study is the short duration of the follow-up period of TAVI and traditional AVR patients. While the choice of 2-months period post-implantation can be judged to be useful in order to highlight the rapid effectiveness of TAVI in improving both LV structure and diastolic function, it should also important to verify whether this improvement could be sustained at longer follow-up. Further studies will be need to analyze this aspect.

In conclusion, our study demonstrates that TAVI could induce a faster recovery of LV geometry than after traditional AVR and the shift from LV concentric remodeling/hypertrophy could be responsible in its turn of a better reduction of LV filling pressure, irrespective of changes in LV afterload.

## Competing interest

The authors declare that they have no competing interests.

## Authors’ contribution

FMC, MG, GDS conceived of the study and participated in its design and coordination, performed the statistical analysis, drafted and revised the manuscript, ED participated in the study design and coordination and performed echo scans, PI, GT and MDN participated in the study design and coordination, FDS, CS and RE participated in the study design, performed and revised the statistical analysis and revised the manuscript. All authors read and approved the final manuscript.
